# The decision-making process for senior cancer patients: treatment allocation of older women with operable breast cancer in the UK

**DOI:** 10.7497/j.issn.2095-3941.2015.0080

**Published:** 2015-12

**Authors:** Jenna L. Morgan, Paul Richards, Osama Zaman, Sue Ward, Karen Collins, Thompson Robinson, Kwok-Leung Cheung, Riccardo A. Audisio, Malcolm W. Reed, Lynda Wyld, on behalf of the Bridging the Age Gap in Breast Cancer Trial Management Team

**Affiliations:** ^1^Academic Unit of Surgical Oncology, University of Sheffield Medical School, Sheffield S10 2RX, UK; ^2^Health Economics and Decision Science, School for Health and Related Research, University of Sheffield, Sheffield S1 4DA, UK; ^3^Center for Health and Social Care Research, Sheffield Hallam University, Collegiate Crescent, Sheffield S10 2BP, UK; ^4^Department of Cardiovascular Sciences, University of Leicester, Leicester LE2 7LX, UK; ^5^School of Medicine, University of Nottingham, Royal Derby Hospital Center, Derby DE22 3DT, UK; ^6^Department of Surgery, University of Liverpool, St Helens Teaching Hospital, St Helens WA9 3DA, UK; ^7^Brighton and Sussex Medical School, University of Sussex, Brighton BN1 9PX, UK

**Keywords:** Frail elderly, breast neoplasms, decision-making

## Abstract

**Objective:**

Up to 40% of women over 70 years with primary operable breast cancer in the UK are treated with primary endocrine therapy (PET) as an alternative to surgery. A variety of factors are important in determining treatment for older breast cancer patients. This study aimed to identify the patient and tumor factors associated with treatment allocation in this population.

**Methods:**

Prospectively collected data on treatment received (surgery *vs.* PET) were analysed with multivariable logistic regression using the variables age, modified Charlson Comorbidity Index (CCI), activities of daily living (ADL) score, Mini-Mental State Examination (MMSE) score, HER2 status, tumour size, grade and nodal status.

**Results:**

Data were available for 1,122 cancers in 1,098 patients recruited between February 2013 and June 2015 from 51 UK hospitals. About 78% of the population were treated surgically, with the remainder being treated with PET. Increasing patient age at diagnosis, increasing CCI score, large tumor size (5 cm or more) and dependence in one or more ADL categories were all strongly associated with non-surgical treatment (*P*<0.05).

**Conclusion:**

Increasing comorbidity, large tumor size and reduced functional ability are associated with reduced likelihood of surgical treatment of breast cancer in older patients. However, age itself remains a significant factor for non-surgical treatment; reinforcing the need for evidence-based guidelines.

## Introduction

A third of new breast cancer diagnoses occur in women aged over 70 years in the UK^1^. The number of women affected will increase over time due to the aging population^2^. The prevalence of co-morbidity and frailty increases with age, resulting in deaths from other causes exceeding breast cancer mortality in older women with breast cancer^3^^,^^4^. Additionally, tolerance of some breast cancer therapies is reduced in this age group^5^^,^^6^. The majority (about 90%) of older women are diagnosed with estrogen receptor (ER) positive disease^3^. Older patients with operable breast cancer may be offered alternative treatment modalities, such as primary endocrine therapy (PET)^7^^,^^8^, where ER positive disease is treated with endocrine therapy alone without surgery. Non-operative management of older breast cancer patients accounts for up to 40% of treatment in the UK^9^.

PET gained popularity in the 1980s for the management of older women after tamoxifen was shown to be effective in this setting^10^ and a succession of randomised controlled trials comparing its efficacy with surgery followed. A subsequent Cochrane review comparing PET with surgery in the over 70s demonstrated superior rates of local control with surgery but no difference in survival rates^11^. However the studies included in the review were small and flawed by modern standards because tumor ER status was not always tested and the age range of the trials included relatively young older women with no documentation of health, fitness or frailty, in fact all patients were deemed fit for surgery under general anaesthesia and therefore frail patients were excluded. A recent review of case series indicated that older frailer women tend to be treated with PET in routine practice and have inferior survival rates as would be expected due to higher other-cause mortality^12^. Since the trials included in the Cochrane review were conducted there have been significant increases in population life expectancy, so it is not clear that whether results of these trials are applicable in contemporary clinical practice.

Recent reports have advocated the use of PET only in the very old or frail^13^. Current national guidelines state that patients with operable breast cancer should be treated with surgery, and not PET, “irrespective of age” unless this is precluded by comorbidities^14^; whilst the International Society of Geriatric Oncology (SIOG) and European Society of Breast Cancer Specialists (EUSOMA) recommend that PET should only be offered to patients with a “short estimated life expectancy (less than 2 to 3 years), who are considered unfit for surgery… or who refuse surgery”^15^. However, as a large number of older women are treated with PET in UK and other countries, it is not clear that this guidance is being followed consistently.

Population cohort studies have been carried out to investigate the factors associated with non-surgical treatment of breast cancer in older women and found that increasing age, comorbidities and worsening scores on activities of daily living (ADL) were all associated with lower rates of surgery^16^. An analysis of English cancer registry data showed that after adjusting for disease characteristics, age and comorbidity, there remained significant variation in the rate of PET use at the hospital level^17^. This may reflect genuine variation in practice, which would contradict current recommendations. However, this analysis could not account for other potential confounding factors such as frailty and cognitive function, which would be expected to influence treatment decision making. There are also limitations concerning data quality such as missing data due to the retrospective and routine nature of data collection. A further confounding factor is the influence of the patient’s preference for one or other option if they are given a choice.

The study presented here aims to identify patient and tumor factors associated with treatment allocation in the older UK breast cancer population, including data on functional status, cognitive function and comorbidities, as well as disease characteristics. A planned interim analysis was undertaken using prospectively collected patient data from a large multicenter UK cohort study.

## Materials and methods

### Study design

This study is part of a prospective observational cohort study of women aged over 70 years diagnosed with operable primary breast cancer in 51 UK breast cancer units between February 2013 and June 2015. Recruitment for this trial is ongoing, and results presented here represent an interim analysis of the first 1,447 patients. Data were collected on patient and tumor characteristics, and treatment type. Data collection time points for the study are at baseline (prior to any treatment being administered), 6 weeks post-treatment, 6 months post treatment, 12 months post-treatment, 18 months post-treatment, and  24 months post-treatment. Analyses are restricted to data collected up to 6 months post-treatment as the outcome of interest was primary treatment allocation.

**Figure 1:**
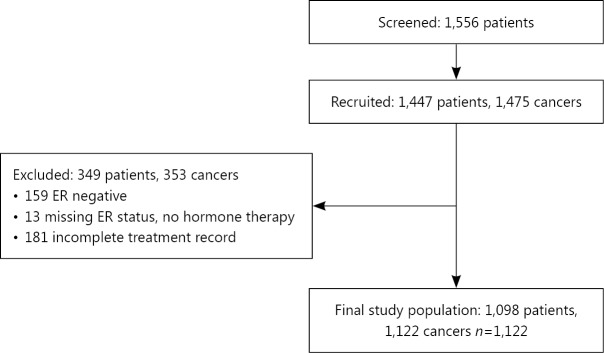
Determining the study population.

Analyses are also restricted to the subgroup of patients with operable ER positive disease at diagnosis. Variables used within the analysis are shown in **Table 1**. Patients with missing treatment data or with known ER negative disease were excluded from the analysis. Patients with missing ER status who did not receive endocrine therapy were assumed to be ER negative and also excluded from the analysis. Bilateral cancers are counted individually. **Figure 1** shows the baseline features of the study population.

**Table 1:** Characteristics of the study population

Characteristics	Total (*n*=1,122)	PET (*n*=242)	Surgery (*n*=880)	*P*
Age (years)				<0.0001
≥70-74		22 (5.5%)	381 (94.5%)	
≥75-79		52 (16.1%)	271 (83.9%)	
≥80-84		65 (30.8%)	146 (69.2%)	
≥85-90		62 (48.4%)	66 (51.6%)	
≥>90		41 (71.9%)	16 (28.1%)	
Median (range)		84 (70-101)	76 (70-94)	
Tumour grade				0.953
≥1		46 (21.7%)	166 (78.3%)	
≥2		144 (22.0%)	510 (88.0%)	
≥3		31 (19.4%)	129 (80.6%)	
Missing		21 (21.9%)	75 (78.1%)	
HER2 status				0.0042
Positive/borderline		17 (12.5%)	119 (87.5%)	
Negative		158 (20.9%)	597 (79.1%)	
Missing		67 (29.0%)	164 (71.0%)	
Nodal status				0.0194
Positive		51 (28.3%)	129 (71.7%)	
Negative		183 (20.0%)	734 (80.0%)	
Missing		8 (32.0%)	17 (68.0%)	
Tumour size				<0.0001
≥<10 mm		15 (12.6%)	104 (87.4%)	
≥10-19.9 mm		64 (17.0%)	312 (83.0%)	
≥20-49.9 mm		126 (24.2%)	395 (75.8%)	
≥≥50 mm		29 (38.7%)	46 (61.3%)	
Missing		8 (25.8%)	23 (74.2%)	
Median size (range) (mm)				
Mini-Mental State Examination (MMSE) score				<0.0001
≥27-30		114 (16.7%)	569 (83.3%)	
≥22-26		37 (27.8%)	96 (72.2%)	
≥0-21		19 (44.2%)	24 (55.8%)	
Missing		72 (27.4%)	191 (72.6%)	
Median MMSE score (range)				
Activities of daily living (ADL) score				<0.0001
Fully independent		128 (15.1%)	720 (84.9%)	
Dependent in 1 or more category		72 (47.4%)	80 (52.6%)	
Missing		42 (34.4%)	80 (65.6%)	
Charlson Comorbidity Index (CCI) score				<0.0001
≥0		71 (13.3%)	462 (86.7%)	
≥1		52 (26.1%)	147 (73.9%)	
≥2		47 (24.0%)	149 (76.0%)	
≥≥3		58 (38.7%)	92 (61.3%)	
Missing		14 (31.8%)	30 (68.2%)	
Median CCI score (range)	<0.0001			

### Statistical analysis

Primary treatment was dichotomised as “surgery” or “PET” according to whether or not the patient received surgery to the primary tumor. Simple logistic regression was used to investigate associations between complete covariates and treatment. The joint effect of patient level factors on the probability of surgical treatment was assessed using multivariable logistic regression.

Variables with missing data cannot be included in standard regression models, but excluding cases with missing values (complete case analysis) can produce biased results where data is not missing completely at random, as well as reducing statistical precision. Missing data on disease characteristics and comorbidity was handled using the method of multiple imputation by chained equations (MICE)^18^. Missing covariate values were imputed using predictive mean matching^19^, and all variables included in the main analysis were incorporated into the imputation models. Twenty-five completed datasets were used for subsequent analysis. Results of the regression models for each dataset were combined using the standard methods described by Rubin^20^. Covariates with over 50% missing data were not included in the regression models.

The performance of the regression model was assessed by considering the receiver-operating characteristic (ROC) curve of the model, along with its corresponding area under the curve (AUC) statistic. This is a measure of the ability of the model to discriminate between individuals treated with surgery and PET^21^. The AUC can range from 0.5 to 1, where a value of 1 indicates perfect discrimination, and a value of 0.5 less indicates a model which performs no better than random chance.

All analyses were conducted using the open source statistical programming language R (version 3.0.1). Multiple imputation was implemented using the “mi” package (version 1.0).

### Ethics and research governance

The study protocols were approved by the UK National Research Ethics Committee (12/LO/1808) and institutional approvals were granted at each site. Written informed consent was obtained from the patient, or patient consultee in the case of patients with cognitive impairment.

## Results

Data were available on 1,122 ER positive breast cancers in 1,098 patients with a treatment record of either primary surgery with adjuvant therapy as deemed appropriate by the treating clinician (“surgery”) or endocrine therapy without surgery (“PET”). Characteristics of the study population can be seen in **Table 1**.

About 78% of the study population were treated surgically, with the remainder being treated with PET. The patients treated with surgery were younger overall than those treated with PET (**Table 1**). The imputation algorithm converged successfully, and exploratory analysis of the distributions of the imputed values did not raise concerns about their validity or plausibility.

**Figure 2:**
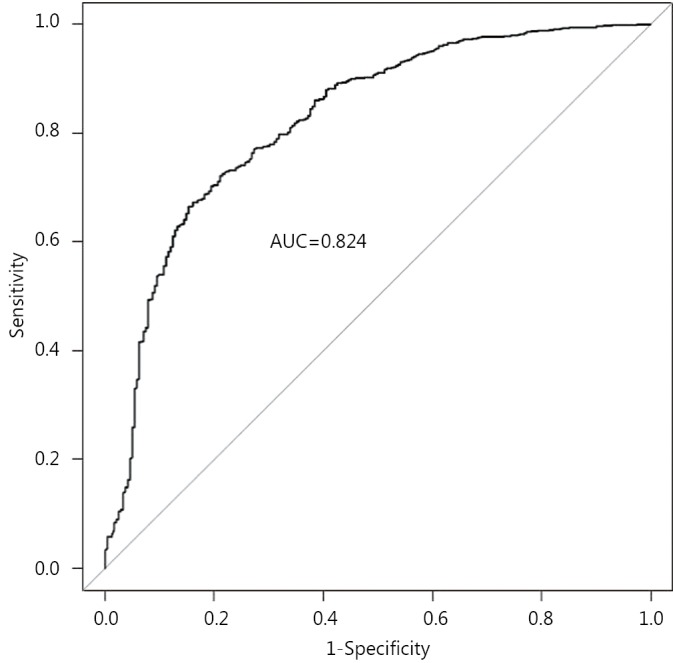
Receiver-operator characteristic (ROC) curve for the multivariable logistic regression model (**Table 2**), with area under the curve statistic (AUC).

The multivariable logistic regression model is shown in **Table 2**. Increased age at diagnosis, increasing levels of comorbidity, large tumor size (5 cm or more) and dependence in one or more ADL categories are all strongly associated with non-surgical treatment (**Table 2**). The ROC curve shows that the model discriminates well between individuals treated with PET and surgery, with the AUC statistic equal to 0.824 (95% CI, 0.792-0.855) (**Figure 2**).

**Table 2:** Multivariable logistic regression of surgical treatment *vs.* non-surgical treatment, conditional on individual patient characteristics

Variable	Odds ratio of having surgery	95% CI	*P*
Age at diagnosis			
70 years	Reference	–	–
Per year over 70	0.84	0.82-0.87	<0.001*
Tumour grade			
1	Reference	–	–
2	0.92	0.58-1.46	0.723
3	1.18	0.63-2.21	0.600
HER2 status			
Negative	Reference	–	–
Positive/borderline	1.71	0.95-3.07	0.075
Nodal status			
Negative	Reference		
Positive	0.85	0.54-1.34	0.495
Tumour size			
<10 mm	Reference	–	–
10-19.9 mm	0.81	0.41-1.61	0.551
20-49.9 mm	0.84	0.56-1.25	0.396
≥50 mm	0.36	0.19-0.70	0.003*
Mini-Mental State Examination (MMSE) score			
27-30	Reference	–	–
22-26	0.89	0.54-1.48	0.658
0-21	0.57	0.26-1.24	0.156
Activities of daily living (ADL) score			
Independent	Reference	–	–
Dependent	0.44	0.28-0.69	<0.001*
Charlson Comorbidity Index (CCI) score			
0	Reference	–	–
1	0.58	0.36-0.95	0.023*
2	0.58	0.37-0.94	0.026*
≥3	0.47	0.28-0.69	<0.001*

Odds ratio >1 indicates increased odds of receiving surgery. * indicates statistical significance at 5% level.

## Discussion

Of the 1,122 ER positive operable breast cancers enrolled into the study to date, 78% were treated with surgery. Age was confirmed as one of the most important factors in determining treatment in older women with operable ER positive breast cancer, with increasing age associated with higher rates of PET, even accounting for other patient and disease characteristics. This is consistent with other studies^7^^,^^22^^,^^23^ despite current UK guidelines, which state that patients should be treated with surgery and appropriate adjuvant therapy “irrespective of age”^14^.

Increasing age was associated with increasing rates of comorbidity and multi-morbidity^24^, this in turn may reduce the survival benefit of more aggressive breast cancer therapies, such as surgery^4^. It was therefore unsurprising that higher levels of co-morbidity in the study population are strongly associated with lower levels of surgical treatment, however, this does not account for the effect seen with age.

Functional dependence in one or more of the ADL categories was strongly associated with non-surgical treatment in this study. Rates of functional dependence also increase with age and have been shown to affect treatment in a small population of older breast cancer patients^22^. Functional dependence has been associated with increased rates of post-operative complications and longer hospital stays in elderly cancer patients^25^, possibly explaining why it has an impact on treatment allocation here.

Additionally, large tumor size (5 cm or more) was also associated with higher rates of PET. Tumors of this size usually mandate a mastectomy^26^, and so these findings may represent patients and clinicians trying to avoid more major surgery.

The rate of surgical treatment was higher in this study than some of the published figures for the UK, which range from 55%-83%^8^^,^^16^^,^^17^^,^^27^^-^^29^. This may result from a recruitment bias as patients within this study had to be recruited prior to commencing treatment, which was logistically more difficult with patients treated with PET as there is a shorter time period between diagnosis and treatment compared with surgery. Another possible bias could be that clinicians may be more likely to ‘follow the guidelines’ knowing that their data were being captured. This will ultimately result in an under-representation of PET patients, and may explain why Lavelle and colleagues found a similar rate of 83% having surgical treatment in their prospective cohort study^16^.

This study is limited by missing data, particularly with relation to ADL and MMSE, which is due, in part of patients opting out of completing the questionnaires. We have used multiple imputation to account for these missing data as it is less prone to bias than other commonly used methods to account for missing data. However, whilst exploratory analysis of the imputed data suggested that the values were plausible, it is not possible to verify the extent to which the distribution of the imputed data accurately represents that of the missing values. By using 25 imputations, uncertainty due to missing data is incorporated into the estimated effects of covariates on the probability of surgery, which mitigates against any small biases due to problems with the imputation model.

The findings of this study confirm existing research showing that age remains a significant predictor of non-surgical treatment after adjusting for disease characteristics and the underlying health status of the patient. However, it remains unclear as to whether or not current practice resulted in sub-optimal outcomes. An attempt to compare outcomes under PET and surgery with adjuvant endocrine therapy using a randomised clinical trial failed due to lack of recruitment, which was explained by an unwillingness on behalf of both patients and clinicians to choose treatment at random^30^. As a result, observational research is needed to investigate the effects of non-surgical treatment in this population. As recruitment and follow up of this cohort matures, it will be possible to investigate the relationship of treatment and individual characteristics with outcomes such as survival and treatment failure. These data will be crucial in the development of evidence based guidance for patients and clinicians to help target treatments effectively.
